# Emotional reactions and hardiness among Russian students during the first wave of the COVID-19 pandemic

**DOI:** 10.1192/j.eurpsy.2022.1281

**Published:** 2022-09-01

**Authors:** V. Rozhdestvenskiy, D. Ivanov, Y. Aleksandrovich, I. Gorkovaya, V. Titova

**Affiliations:** Saint Petersburg State Pediatric Medical University, Department Of Psychosomatics And Psychotherapy, Saint Petersburg, Russian Federation

## Abstract

**Introduction:**

The COVID-19 pandemic provoked emotional reactions in Russian student populations. Hardiness is seen as a personality predisposition that increases individuals’ resilience to stress.

**Objectives:**

The study aimed to determine the severity of depression, anxiety, stress, and various components of hardiness in Russian students. In addition, the correlations between emotional reactions and hardiness components were also analysed.

**Methods:**

Data collection was carried out between 29 May and 06 July 2020. A total of 129 medical and non-medical students participated in the study. The DASS-21 was used to measure depression, anxiety, and stress levels, while the Personal Views Survey-III examined hardiness. Both questionnaires were adapted for use in Russia.

**Results:**

We found that medical students were less likely to be depressed than non-medical students (M = 4.03 and M = 6.01 respectively, p < 0.05). Medical students had higher levels of the component of hardiness such as commitment (M = 20.95 and M = 18.43 respectively, p < 0.05). In both groups, all hardiness components have negative relationships with depression, anxiety, and stress, but in the medical group control is associated only with depression (r_x_ = -0.446, p < 0.01), whereas the other group also has associations with anxiety (r_x_ = -0.356, p < 0.01) and stress (r_x_ = -0.407, p < 0.01).

**Conclusions:**

Hardiness was negatively related to depression, anxiety, and stress in a pandemic setting. Medical students were more adaptable to the pandemic than non-medical students.

**Disclosure:**

No significant relationships.

